# Excess mortality in refugees, internally displaced persons and resident populations in complex humanitarian emergencies (1998–2012) – insights from operational data

**DOI:** 10.1186/s13031-016-0082-9

**Published:** 2016-07-20

**Authors:** Peter Heudtlass, Niko Speybroeck, Debarati Guha-Sapir

**Affiliations:** Institut de Recherche Santé et Société, Université catholique de Louvain, Brussels, Belgium

**Keywords:** Complex humanitarian emergencies, Mortality, Displacement, IDPs, Refugees

## Abstract

**Background:**

Complex humanitarian emergencies are characterised by a break-down of health systems. All-cause mortality increases and non-violent excess deaths (predominantly due to infectious diseases) have been shown to outnumber violent deaths even in exceptionally brutal conflicts. However, affected populations are very heterogeneous and refugees, internally displaced persons (IDPs) and resident (non-displaced) populations differ substantially in their access to health services. We aim to show how this translates into health outcomes by quantifying excess all-cause mortality in emergencies by displacement status.

**Methods:**

As standard data sources on mortality only poorly represent these populations, we use data from CEDAT, a database established by aid agencies to share operational health data collected for planning, monitoring and evaluation of humanitarian aid. We obtained 1759 Crude Death Rate (CDR) estimates from emergency assessments conducted between 1998 and 2012. We define excess mortality as the ratio of CDR in emergency assessments over ‘baseline CDR’ (as reported in the World Development Indicators). These death rate ratios (DRR) are calculated separately for all emergency assessments and their distribution is analysed by displacement status using non-parametric statistics.

**Results:**

We found significant excess mortality in IDPs (median DRR: 2.5; 95 % CI: [2.2, 2.93]) and residents (median DDR: 1.51; 95 % CI: [1.47, 1.58]). Mortality in refugees however is not significantly different from baseline mortality in the host countries (median DRR: 0.94, 95 % CI: [0.73, 1.1]).

**Conclusions:**

Aid agencies report the highest excess mortality rates among IDPs, followed by resident populations. In absolute terms however, due to their high share in the total number of people at risk, residents are likely to account for most of the excess deaths in today’s emergencies. Further research is needed to clarify whether the low estimates of excess mortality in refugees are the result of successful humanitarian interventions or due to limitations of our methods and data.

## Background

A complex humanitarian emergency is a “multifaceted humanitarian crisis in a country, region or society where there is a total or considerable breakdown of authority resulting from internal or external conflict and which requires a multi-sectoral, international response” [1].

Affected populations can broadly be classified as refugees, internally displaced persons (IDPs) or resident (that is non-displaced) populations [2]. Refugees flee their countries of origin and are under special protection by the United Nations High Commissioner for Refugees (UNHCR). IDPs leave their homes and livelihoods behind but have not (yet) fled the country and might live among the host population or settle in IDP camps.

By the end of 2013, the global number of refugees was reported to be 16.7 m - the vast majority of them (86 %) hosted by developing countries - and the number of IDPs 33.3 m [3]. The size of resident populations affected by complex humanitarian emergencies is more difficult to determine, as there is no systematic data collection. This also reflects the absence of a specific lobby organisation for non-displaced populations - a role assumed by the UNHCR for refugees or, somewhat less prominently, by the Internal Displacement Monitoring Centre (IDMC) for IDPs. Resident populations affected by conflict are likely to outnumber refugees and IDPs by far. According to an estimation based on data from the Armed Conflict Location and Event Dataset [4], 87 % of all people affected by complex emergencies in 2012 were residents [5].

Not only their number, but also their specific needs make people affected by complex emergencies one of the top priorities on the global public health agenda [6]. Lack of access to food, water, shelter, sanitation and medical care cause a substantial burden of excess mortality due to preventable infectious diseases [2]. These non-violent deaths can easily outnumber violent deaths, even in particularly brutal conflicts such as the 2003 genocide in Darfur [7].

However, there are no systematic, global statistics on the burden of excess mortality carried by IDPs, refugees and residents who are affected by complex humanitarian emergencies. This is mainly because these populations are chronically underrepresented in standard sources of demographic and epidemiological datasets used for public health planning such as censuses, vital registration systems or even large-scale studies like the Demographic and Health Surveys (DHS). For instance, in the DHS Colombia 2010, investigators deviated from the random sample and replaced conflict-affected units by non-affected ones [8]. Similarly, security concerns prevented the surveyors to visit census enumeration areas in Ethiopia’s Somali region that were randomly selected for the DHS Ethiopia 2011 and estimates may not be representative for this region [9].

For this reason, humanitarian aid agencies resort to collecting their own data when planning, monitoring and evaluating public health interventions in complex emergencies. They typically conduct fairly regular small-scale health assessments, limited to their organisation’s specific mandate and geographic scope. The availability of this kind of operational data is growing with the number and size of internationally active humanitarian agencies. Moreover, data quality is gradually improving, even though there still exist areas of further development, particularly with regard to the assessment of mortality indicators [10–12].

In 2003, a multi-agency initiative established the complex emergency database (CEDAT), a global survey repository that centralises and consolidates routinely collected mortality, health and nutrition data from aid agencies [13]. In this study, we use the CEDAT database to quantify excess mortality in IDPs, refugees and resident populations in complex humanitarian emergencies.

## Methods

CEDAT contains data from 3186 different surveys, conducted by more than 40 different humanitarian organisations in 54 different countries and territories between 1998 and today [14].

CEDAT is a repository of survey results: survey reports from contributing aid agencies are searched for relevant health, nutrition and mortality estimates which are entered onto an electronic database. Death rates reported in the CEDAT database are extracted from household surveys in which mortality is typically estimated retrospectively for a specific recall period; based on records of deaths, births and the number of people leaving and joining the household [15]. Death rates are calculated as the number of deaths during the recall period divided by the number of people at risk after half of the recall period. They are reported for different age groups and using various units for the person-time spent at risk.

At the time of the data download for this study, CEDAT included 4498 mortality estimates for different age groups and in various formats. In this analysis, we are only looking at estimates of the Crude Death Rate (CDR), which is a measure of all-cause mortality in all age groups. CDR is our choice because it is the most comprehensive mortality indicator in CEDAT. Other reported indicators typically represent sub-groups of the CDR population (for instance Under Five Death rate or Infant Death Rate). We include estimates that were reported using units of “deaths per 10 000 per day” or any estimates that can be converted into the same format (i.e. “deaths per 1000 per year”). This is the recommended format to be used in public health emergencies [15, 16]. We excluded one observation for which the year of the assessment was missing and retrieved 2072 CDR estimates from complex emergencies between 1998 and 2012 in 42 different countries that fulfil these criteria. (For lists of mortality estimates by year and country see Tables 1 and 2.) Note that in the case of refugees, ‘country’ designates the host country.

**Table 1:** Number of Crude Death Rate estimates in CEDAT by country and population group

	Resident	IDP	Refugee	Mixed	Other
Afghanistan	22	4	0	5	9
Angola	19	25	0	19	12
Bangladesh	8	0	1	0	0
Burundi	19	0	0	1	0
Cameroon	0	0	1	0	0
Central African Republic	9	0	0	1	0
Chad	13	2	39	8	0
Colombia	0	0	0	1	0
Congo	0	0	3	0	3
Cote d’Ivoire	1	0	0	0	0
Democratic Republic of the Congo	365	4	11	21	6
Djibouti	1	0	2	0	0
Eritrea	1	1	0	0	0
Ethiopia	258	4	32	5	1
Ghana	0	0	2	0	0
Guatemala	2	0	0	0	0
Guinea	6	0	4	0	0
Haiti	33	0	0	0	0
Iraq	4	0	0	0	0
Kenya	61	4	10	6	0
Liberia	6	15	1	3	4
Malawi	56	0	0	0	0
Mali	7	0	0	0	0
Mauritania	9	0	1	0	0
Myanmar	9	0	0	0	0
Namibia	0	0	1	0	0
Nepal	4	0	0	0	0
Niger	95	0	0	0	0
Nigeria	1	0	0	0	0
Pakistan	2	0	10	0	0
Rwanda	0	0	2	0	0
Sierra Leone	10	16	0	1	3
Somalia	168	37	0	15	3
South Sudan	2	0	3	3	2
Sudan	154	91	0	130	39
Tajikistan	9	0	0	0	0
Tanzania	0	0	5	0	0
Timor-Leste	1	0	0	0	0
Uganda	3	41	4	9	3
Yemen	2	0	6	0	0
Zambia	0	0	8	0	0
Zimbabwe	9	0	0	0	0

**Table 2:** Number of Crude Death Rate estimates in CEDAT by year and population group

	Resident	IDP	Refugee	Mixed	Other
1998	3	0	0	0	0
1999	0	1	0	1	0
2000	31	16	1	18	4
2001	29	8	1	20	2
2002	79	23	3	12	3
2003	62	21	46	14	14
2004	109	30	6	23	16
2005	117	35	10	35	11
2006	161	12	11	15	5
2007	179	25	9	22	3
2008	137	19	29	28	10
2009	220	10	17	4	5
2010	113	19	7	23	6
2011	118	25	2	9	4
2012	11	0	4	4	2

**Fig. 1:**
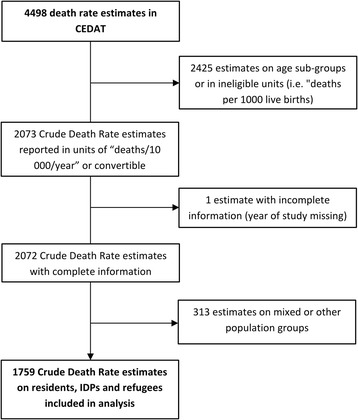
Flow chart

Of these death rates, 1369 (66.1 %) were estimated for residents, 244 (11.8 %) for IDPs, 146 (7 %) for refugees, 85 (4.1 %) for other population groups (for instance returnees/resettled populations and nomads), and 228 (11 %) for populations mixed in various proportions of the aforementioned groups (mixed populations). We excluded data from other population groups as well as from mixed populations, as we do not have reliable information on the mixing proportions. Thus, a total of 1759 death rate estimates from either resident, IDP or refugee populations remained for analysis (Fig. 1).

The study populations of the emergency mortality assessments range from single camps, groups of villages, districts and health zones to states or provinces; the median population size varies between 16 939 people per study in refugees, 63 210 people per study in IDPs and 151 663 people per study resident populations. Median sample sizes range from 930 subjects (residents) to 2756 subjects (refugees). Median recall periods are 91 days for all three population groups (Table 3). Typically, the geographic scope of individual assessments is not limited by specific administrative boundaries but rather by the aid agency’s area of activity.

**Table 3:** Population sizes, sample sizes and recall periods

	IDP	Refugee	Resident
Population size: median (NAs/n, IQR)	63210 (123/244, 16220–101700)	16940 (43/146, 12000–30490)	151700 (520/1369, 80000–271200)
Sample size: median (NAs/n, IQR)	985.5 (106/244, 766.2–4300)	2756 (92/146, 793–4054)	930 (586/1369, 632–3644)
Recall period (in days): median (NAs/n, IQR)	91 (49/244, 90–122)	91 (69/146, 91–110)	91 (218/1369, 90–98)

The concept of excess mortality in humanitarian emergencies is based on the idea of a certain level of all-cause mortality that is assumed to be unrelated to the emergency, often referred to as baseline mortality [15]. Excess mortality can then be defined as any difference between observed mortality and baseline mortality. Here, we look at the rate ratio of emergency death rates over baseline death rates instead of the absolute difference between these in order to ensure that excess mortality estimates are comparable across countries and years with differing baseline mortality.

For each individual emergency assessment, we use the CDR estimate from the World Bank’s World Development Indicators (WDI) for the same country and year as baseline reference [17]. The implicit assumption is that the surveyed population in the emergency assessments have a demographic profile similar to the rest of the country’s population, as CDRs are not age-standardised and therefore, all other things being equal are usually higher in older populations than in younger ones. We believe using WDI data is a conservative approach, as these estimates are likely to include at least parts of the affected populations and are therefore overestimating mortality in non-affected populations (and thus underestimating excess mortality, the indicator of interest). Moreover, the dataset is comprehensive enough to cover the study period and combines information from multiple sources. To make it comparable, we converted the WDI data from the original format (deaths/1000/year) to the one that is recommended for use in humanitarian emergencies (deaths/10 000/year).

We group observations by displacement status, calculate median death rate ratios and estimate confidence intervals for these medians using bootstrap methods. We use medians instead of means because medians are less influenced by outliers and a more meaningful statistics of central tendency in skewed distributions (death rates and death rate ratios usually do not follow a normal distribution). We compare the medians to find out whether excess mortality in our data is determined by whether the population was categorised as resident, IDP or refugee. We test whether observed differences are statistically significant by calculating the Kruskal-Wallis test with three groups (IDP, resident and refugee) and pairwise, two-sided Wilcoxon rank-sum tests with a confidence level of 0.95.

In order to explore whether potential differences in the death rate ratios are not simply due to country effects, we conduct a sensitivity analysis and repeat the analysis for the subsets of each of the countries in the sample for which we have observations on each of the three population groups (Chad, DR of the Congo, Ethiopia, Kenya, Liberia and Uganda). If displacement status is indeed a determinant for differences in the death rate ratios, we would expect to see similar difference in these subsets.

All analysis was done in R version 3.0.1 [18].

## Results

**Fig. 2:**
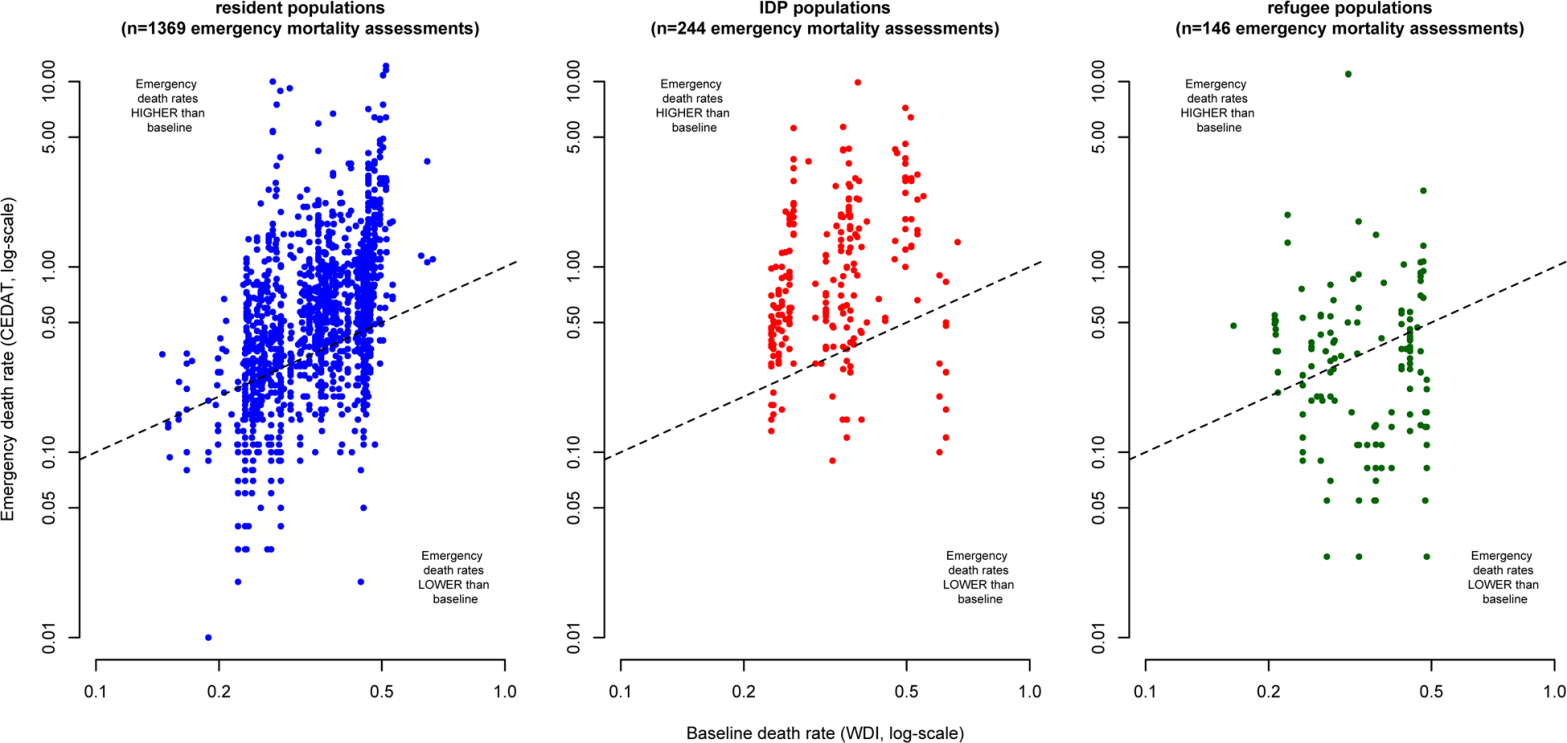
Crude Deaths Rates from emergency mortality assessments compared to baseline death rates, by displacement status

Figure 2 shows death rates observed in humanitarian emergencies plotted against their corresponding baseline values. Note that some extreme values are outside the plotting area and that observations with zero values cannot be plotted due to the log-scale. The closer observations are to the diagonal (dashed lines) in the scatterplots, the more equal are emergency and baseline death rates. Observations above the diagonal indicate excess mortality and observations below the diagonal situations in which observed death rates are lower than baseline death rates.

As emergency mortality assessments are sampling surveys that are based only on a relatively small number of households, we do expect some degree of dispersion of observations above and below the diagonal due to sampling error, even in a situation where there is no true difference between emergency and baseline death rates.

However, in the case of resident populations and IDPs, the mass of the distribution of CDR estimates is located above the diagonal, indicating a tendency towards excess mortality. On the other hand, observations from refugee populations appear to be equally dispersed below and above the diagonal.

**Fig. 3:**
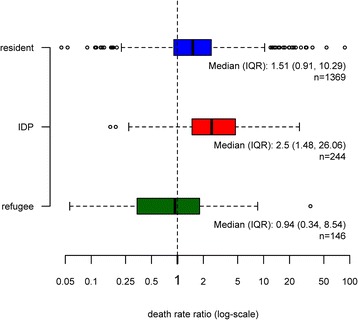
Boxplot of death rate ratios (emergency death rates over baseline death rates), by displacement status

Another way to look at this pattern is by analysing the frequency distributions of death rate ratios (Fig. 3). Again, zero values cannot be plotted due to the log-scale, but are included in the calculation of all statistics. A death rate ratio of 1 implies that the mortality observed in the emergency assessment is equal to the baseline mortality. Almost three quarters of the assessments in resident populations showed mortality that was higher than baseline – in 50 % of them more than 1.5 times higher. In IDP populations, death rate ratios are even larger and 50 % of the assessments show ratios of 2.5 or more. As to refugees, we observe a similar pattern as in Fig. 3: about half of the assessments resulted in death rate ratios lower than 1 and half of them in ratios larger than 1, indicating no systematic difference between emergency mortality rates among refugees and baseline death rates in host countries.

We estimate that median death rate ratios in resident populations are 1.51 (95 % CI: [1.47, 1.58]), in IDPs 2.5 (95 % CI: [2.2, 2.93]) and in refugees 0.94 (95 % CI: [0.73, 1.1]), which means that the death rate ratios are significantly different from 1 in residents and IDPs but not in refugees. The Kruskal-Wallis test shows that the samples of IDP, refugee and resident DDRs do not come from the same distribution (X^2^ = 122.7, df = 2, *p*-value < 0.001). Furthermore, the two-sided Wilcoxon rank-sum tests (confidence level: 0.95) for each pair of the three population groups confirm that all differences between the distributions of death rate ratios in residents, IDPs and refugees are statistically significant (Table 4).

**Table 4:** Wilcoxon rank sum test results

Group 1	Group 2	Median_1_	Median_2_	n_1_	n_2_	Test statistics (W)	*P*-value (two-sided)
Residents	refugees	1.51	0.94	1369	146	133941	< 0.001
Residents	IDPs	1.51	2.5	1369	244	111499	< 0.001
IDPs	refugees	2.5	0.94	244	146	28225	< 0.001

**Fig. 4:**
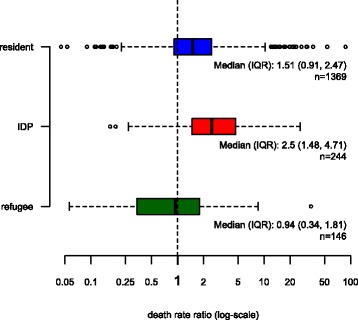
Sensitivity analysis 1: Chad

**Fig. 5:**
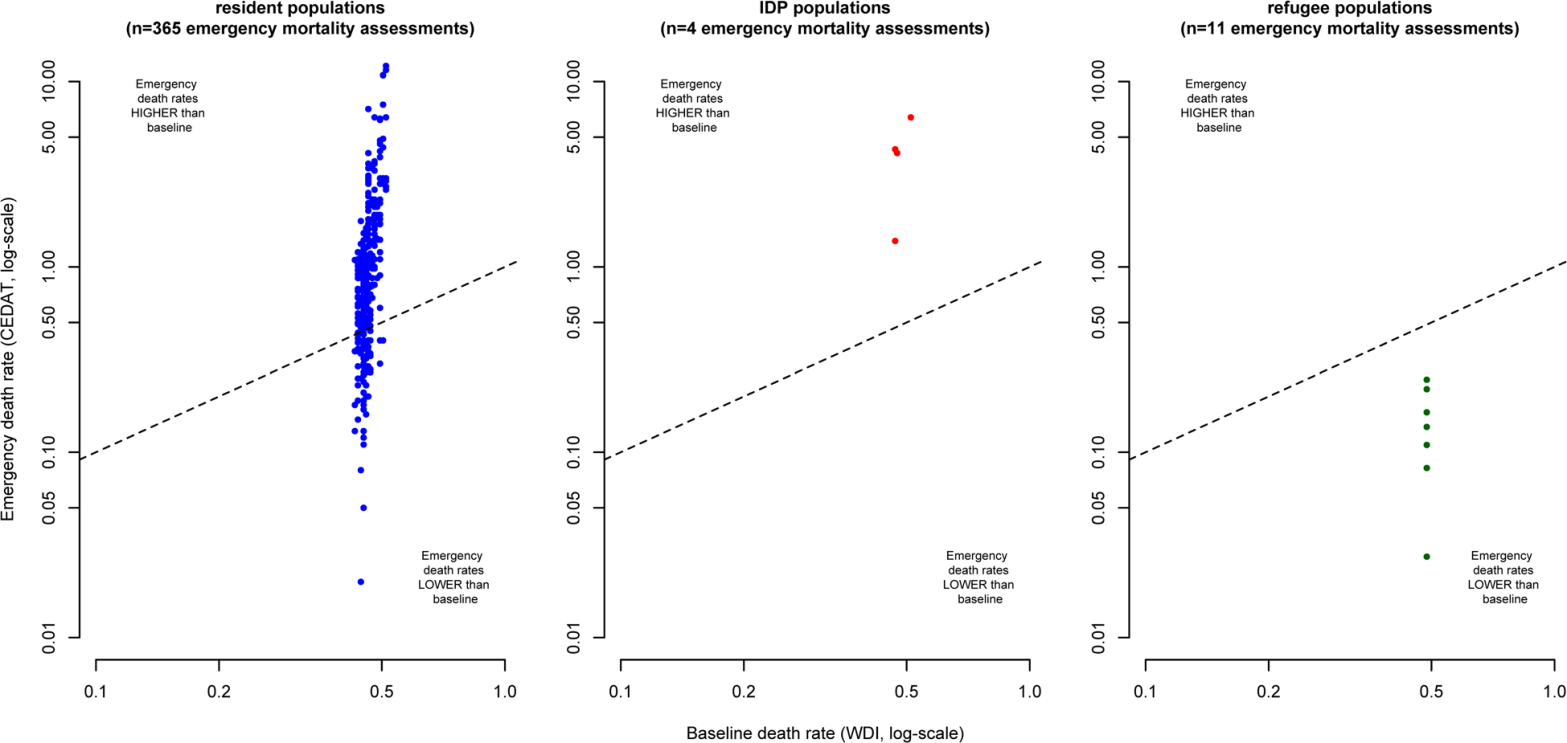
Sensitivity analysis 2: DR of the Congo

**Fig. 6:**
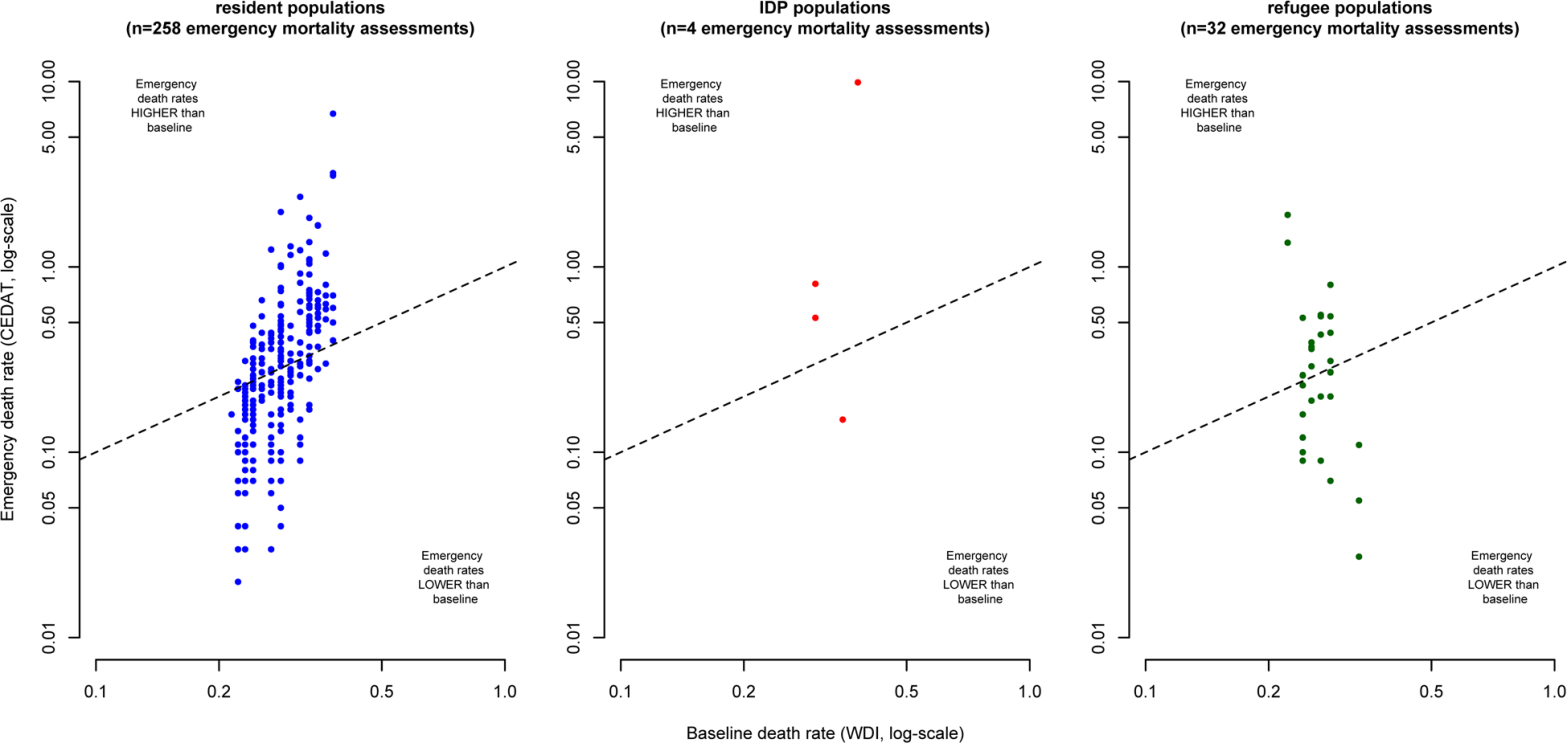
Sensitivity analysis 3: Ethiopia

**Fig. 7:**
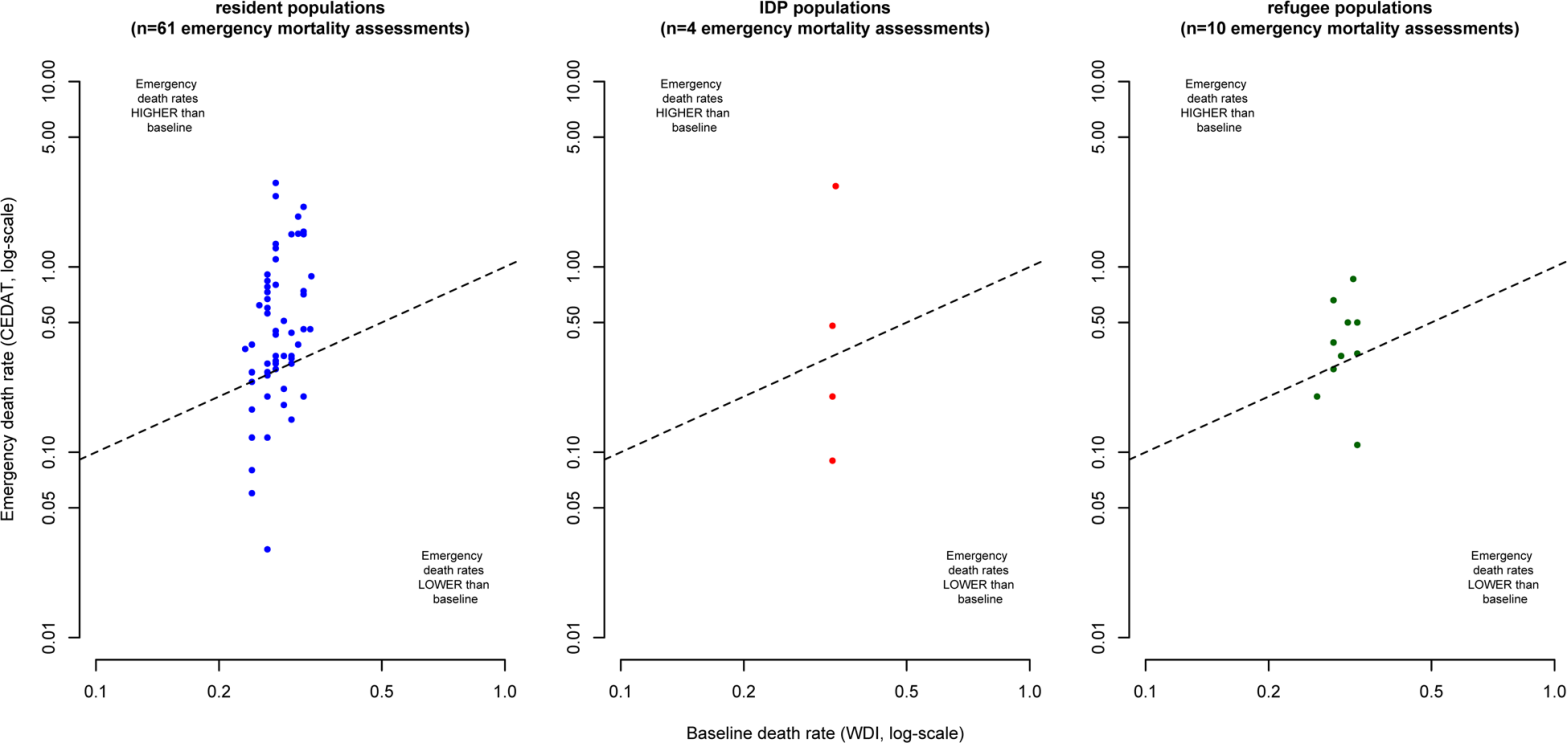
Sensitivity analysis 4: Kenya

**Fig. 8:**
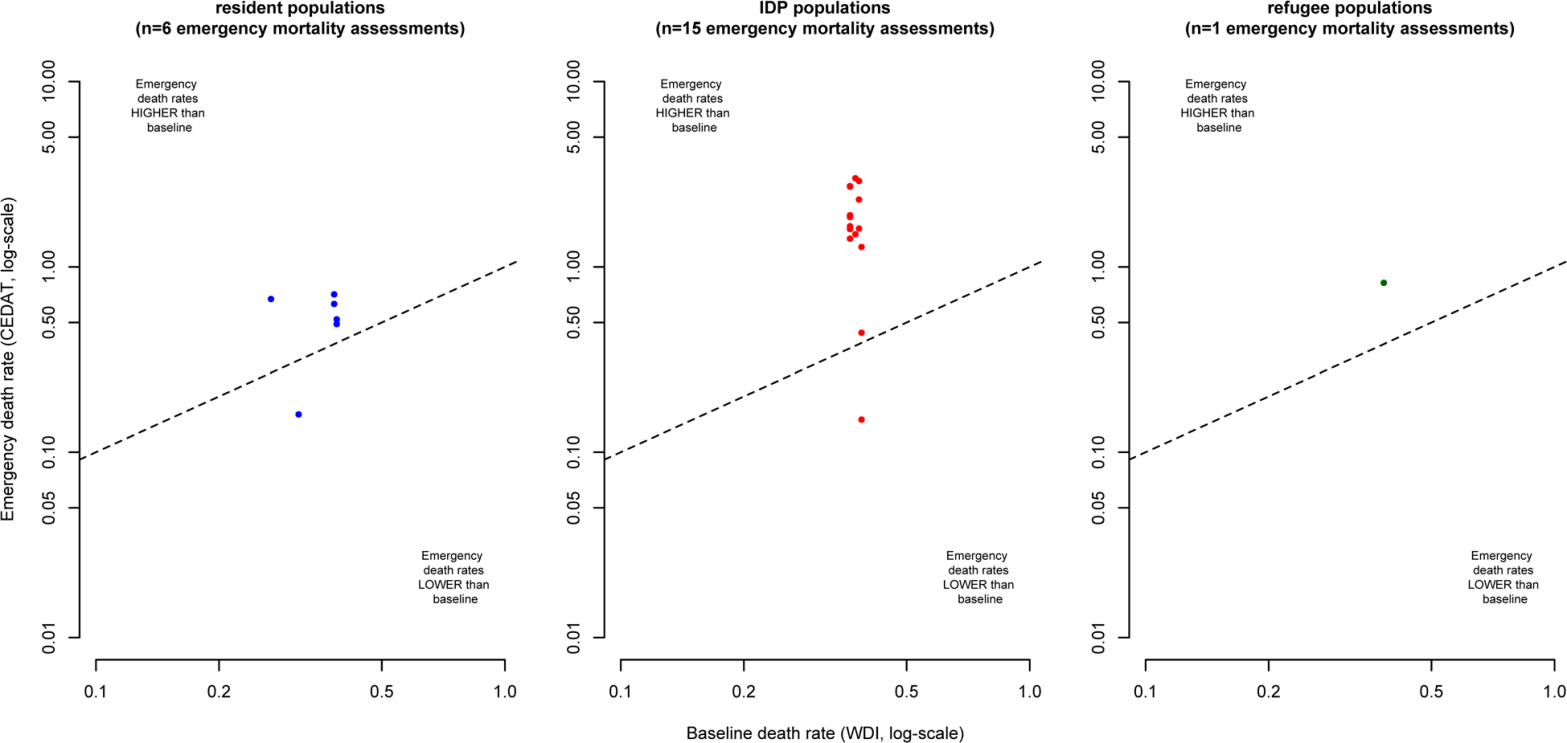
Sensitivity analysis 5: Liberia

**Fig. 9:**
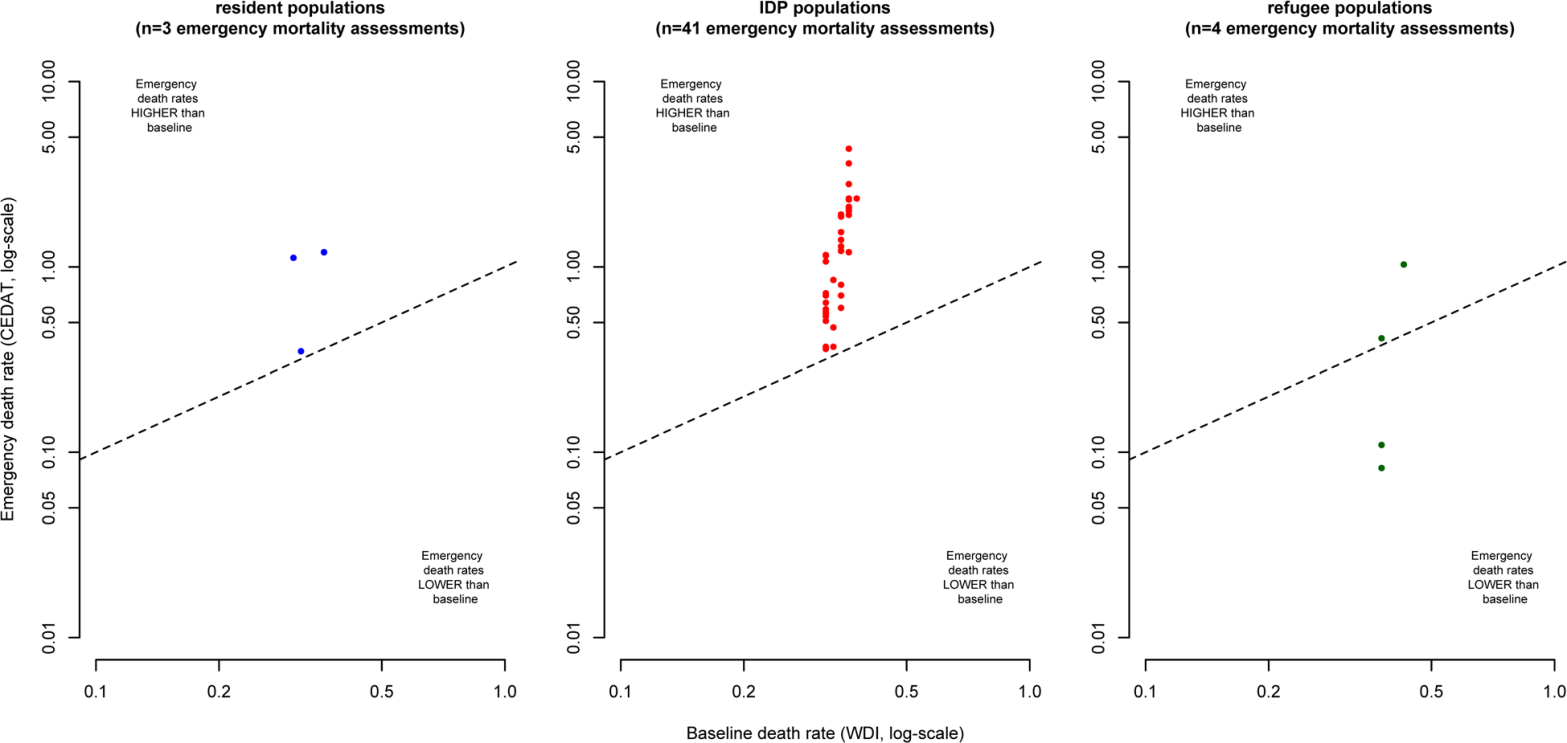
Sensitivity analysis 6: Uganda

When reproducing the plots of Fig. 3 individually for each of the six countries where we have observations on all three population groups, we observe similar pattern as with the complete dataset, even though the number of observations is relatively low, particularly with regards to surveys from IDPs and refugees (Figs. 4, 5, 6, 7, 8 and 9).

## Discussion and conclusions

Most of the data used in this analysis originate from the large humanitarian catastrophes in Sudan, Somalia, DRC and Ethiopia in the mid- and late 2000s and this limits the generalisability of our findings. Although we expect similar mortality pattern in the ongoing crises in Syria, Iraq, Gaza and other places in the world, there are only few recent data in CEDAT available yet. While data sharing among humanitarian agencies has improved, it usually takes at least about one or two years for survey results to be disseminated and available to the public.

Our analysis is at risk of bias at two levels: at the individual survey level and at the meta-analysis level. At the individual survey level, there is for instance the risk of recall or survival biases. These biases are discussed in detail by Checchi and Roberts [12] and can lead to either overestimation or underestimation of mortality rates. Our conclusions are based on the assumption that any biases at survey level are not systematic, that is in some surveys true mortality rates are overestimated and in others true mortality rates are underestimated, but the reasons are not related to the displacement status of the population.

The more crucial assumption underlying our conclusions is that there is no bias at the meta-analysis level, in particular that the surveys in our analysis can be considered a representative sample of eligible surveys and that there is no selection bias.

There is no official register for emergency needs assessments, such as they exist for instance for clinical studies, and it is therefore difficult to determine the share of potentially eligible surveys that are not included in CEDAT and therefore missing in our analysis. To our best knowledge, the main reason that potentially eligible surveys are not included in CEDAT and therefore missing in our analysis is that these surveys are conducted by organizations not collaborating with CEDAT (a list of organizations that work with CEDAT can be found here: http://cedat.be/partners). CEDAT partner organizations represent a wide range of humanitarian agencies and we have no reason to believe that whether or not an organization is collaborating with CEDAT is associated with the level of mortality among their beneficiaries or the DDR.

Some surveys might be missing even though there is an agreement to collaborate with CEDAT: for instance, contacts in collaborating organizations might change, organizations might forget to submit surveys, or some survey reports might never be finalized and so on. These reasons for missingness limit the statistical power of our analysis but do not necessarily introduce a bias. However, a bias might be introduced if any given survey’s likelihood of inclusion in CEDAT (and consequently in our analysis) depends on the mortality rate. For instance: most of the studies in CEDAT are needs assessments and it might be reasonable to assume that if the assessment finds high mortality rates (high needs), humanitarian agencies have a particular interest in disseminating the results to attract more funds for their relief operations. If this was true, we would overestimate mortality in IDPs, refugees and residents in complex emergencies. However, we believe that the risk associated to this bias is fairly low: mortality surveys are quite expensive and organizations will be held accountable by their donors to deliver and disseminate results. Moreover, even if we overestimated mortality rates, there is no reason to believe that the size of this bias differs between IDPs, refugees and residents and therefore the bias would not impact our conclusions with regard to the relative pattern of excess mortality between these population groups.

This analysis would also be biased if agencies were more likely to conduct mortality surveys in particular locations and time periods, for instance, where they expect mortality to be high in order to attract more relief funds. From our experience, this bias is not very likely because small-scale surveys in CEDAT are routinely done at all stages (assessment, monitoring and evaluation) of relief operations. If an agency’s intention was to document high levels of mortality for advocacy purposes, a small-scale survey would probably not be the first choice. As useful as these surveys can be in a meta-analysis of mortality, individually they are quite limited in scope and detail, as mortality is just one of many health indicators being assessed. Even if we cannot, of course, completely exclude the possibility of such a bias, we believe that as in the case of missing surveys, it would probably affect surveys from IDPs, residents and refugees in the same way.

Despite improvements in quality of publicly accessible and comparable health data from humanitarian emergencies, for many of the estimates we still lack sufficient information needed to perform more robust meta-regressions, such as sample sizes, design effects for cluster samples, numbers of deaths (instead of aggregated rates), length of individual recall periods and more precise information on the study area/population.

The surveys were categorised into IDP, refugee, resident and mixed populations by the aid agencies that have conducted the original research. We were not able to validate the quality and consistency of this categorisation. Also, we had to exclude mixed populations from the analysis as we do not have sufficient information on mixing proportions.

Most importantly, this is an observational study and we only show that differences in excess mortality are associated with population status. We do not show causality. For instance, in CEDAT, excess mortality generally appears to be lower in surveys on refugees than in surveys on IDPs, but we cannot say with certainty whether this is due to the fact that they are refugees and not IDPs. Possibly, some confounding factor, influencing both mortality and displacement status, might explain this association. From the (admittedly few) countries that we were able to include in our sensitivity analysis, it seems though that at least the country where the survey takes place is unlikely to be such a confounder.

There is a high degree of variability in death rates between individual surveys in all three population groups. Further research is needed to explain this variability: What part of it can be explained by sampling error? What other factors play a role?

Above limitations notwithstanding, we believe this analysis provides evidence of substantial excess mortality in humanitarian emergencies and that displacement status of affected population is an important determinant of this excess mortality. When compared to baseline data, aid agencies report the highest death rates among IDPs, with observed deaths rates more than twice the baseline, followed by death rates in resident populations. Strikingly, we do not observe any significant excess mortality when comparing refugee death rates to the death rates in their host communities. This could be due to limitations in our analysis: Refugee populations might be healthier and/or younger than host populations - possibly due to some kind of healthy migrant effect - but we are unable to control for this as we do not have access to age standardized data and baseline data.

However, if there is indeed no significant excess mortality in refugees, this might show that aid agencies can successfully prevent mortality if they have access to affected populations and sufficient resources. Being protected by the UNHCR and at least geographically separated from the origin of the emergency, refugees can arguably be more easily assisted by aid agencies. They generally benefit from better access to food, shelter and health services than IDPs or resident populations, who are much more difficult to be identified and reached [2].

We believe that there is a need to improve the collection of standardized epidemiological data on all people affected by complex humanitarian emergencies, particularly on hard-to-reach populations such as IDPs and affected residents. Our estimates suggest that an enormous number of lives could be saved if mortality could be brought down to baseline levels in IDP and resident populations. Although IDPs have a higher death rate ratio, the potential benefit in terms of the absolute number of lives saved is possibly greater in resident populations which outnumber IDPs by far.

## Abbreviations

CDR: crude death rate
CEDAT: complex emergency database
CHE: complex humanitarian emergency
DRR: death rate ratio
IDMC: internal displacement monitoring centre
IDP: internally displaced person
UNHCR: United Nations High Commissioner for Refugees
WDI: world development indicators
